# The expression changes of transcription factors including *ANKZF1*, *LEF1*, *CASZ1*, and *ATOH1* as a predictor of survival rate in colorectal cancer: a large-scale analysis

**DOI:** 10.1186/s12935-022-02751-3

**Published:** 2022-11-07

**Authors:** Manizheh Sajadi, Mohammad Fazilti, Habibollah Nazem, Mohammad Mahdevar, Kamran Ghaedi

**Affiliations:** 1 Department of Biochemistry, Faculty of Science, Payame Noor University, Isfahan, Iran; 2 Department of Biochemistry, Payame Noor University, P.O.Box 19395-4697, Tehran, Iran; 3 Genius Gene, Genetics and Biotechnology Company, Tehran, Iran; 4 Department of Cell and Molecular Biology and Microbiology, Faculty of Biological Science and Technology, University of Isfahan, Isfahan, Iran

**Keywords:** Gene expression, Survival rate, Biomarkers, Master genes

## Abstract

**Introduction:**

Transcription factors (TFs) are essential for many biological processes and regulate the expression of several genes. This study’s objective was to analyze the abnormalities in TF expression, their impact on patient prognosis, and related pathways in colorectal cancer (CRC).

**Method:**

The expression alterations of all TFs were investigated using the cancer genome atlas and GSE39582 data. Clinical data were also used to study the association between TFs expression and patient prognosis through the Cox regression test, and a predictive model of CRC patient survival was constructed based on TFs expression. Co-expression network was used to discover TF-related pathways. To validate the findings, the RT-qPCR method was applied to CRC samples and adjacent normal tissue.

**Results:**

The findings revealed that *ANKZF1, SALL4, SNAI1, TIGD1, LEF1, FOXS1, SIX4*, and *ETV5* expression levels increased in both cohorts and were linked to the poor prognosis. *NR3C2, KLF4, CASZ1, FOXD2, ATOH1, SALL1*, and *RORC* expression, on the other hand, exhibited a significant decrease, and their increase was related to the good prognosis of patients. The patient mortality risk model based on expression of mentioned TFs revealed that, independent of clinical characteristics, the expression of *ANKZF1*, *LEF1*, *CASZ1*, and *ATOH1* could accurately predict patient survival rates. According to the co-expression network, increased transcription factors were linked to metastatic pathways, while decreasing TFs were involved to apoptotic pathways. RT-qPCR findings showed that *FOXS1* expression was markedly overexpressed in CRC samples. However, in CRC samples, the expression of CASZ1 decreased.

**Conclusion:**

In CRC, TFs expression of *ANKZF1*, *LEF1, CASZ1* and *ATOH1* are deregulated, which are associated with prognosis in patients. According to our findings, changes in the expression of the mentioned TFs have the potential to be considered diagnostic and prognostic biomarkers for CRC patients.

## Introduction

Colorectal cancer (CRC) is one of the most frequent cancers and, after lung cancer, was the second greatest cause of death from cancer in 2020 [1]. According to molecular studies, several genes’ expression is changed in this cancer, and these changes are connected to an elevation and progression of malignancy in CRC [2]. Gene level can potentially be used as a biomarker to predict the prognosis of CRC patients [3]. As a result, finding the altered gene expression in CRC can be a highly useful therapeutic and diagnostic goal.

A subset of genes called transcription factors (TFs) are referred to as master genes because changes in their expression and activity have an impact on the level of other genes. In reality, one TF has the ability to control the expression of numerous genes simultaneously and regulate key cellular functions. The expression and activity of some TFs, like TP53, are significantly altered in cancer cells, which can either promote or decrease the aggressiveness and proliferation of cancer cells. However, investigations have indicated that the expression of transcription factors such Snail, *TTF1*, and *ZEB1* are linked to CRC growth and metastasis [4–6]. Increased expression of a variety of transcription factors, including *PROX1* and *GTF3*, is also linked to a poor prognosis in CRC patients [7, 8]. As a result, transcription factor expression in CRC becomes dysregulated, which is related to disease progression and malignancy, and transcription factors may be helpful molecules for targeted treatment and diagnosis.

TFs can change the expression of a large number of their downstream genes. So, altering a large number of disease-related genes at once can be accomplished by targeting a disease-related TF. Additionally, TFs expression changes and their link to CRC patient mortality rates have received less attention, and many of their functions are still unknown. The goal of this study was to look at the expression change of all transcription factors in CRC patients to determine whether there was any correlation between them and patient survival. The linked pathways were also investigated, and the results were confirmed using CRC tissue samples and the RT-qPCR approach. The expression of all transcription factors, as well as their connection with patient survival, were assessed using the cancer genome atlas (TCGA) and GSE39582 data. Transcription factors with oncogenic and tumor suppressor potential were also discovered, and a model for patient mortality risk was presented based on their expression.

## Material and method

### Data collection and preprocessing

The differential expression of transcription factors in colon cancer was assessed using transcriptome data from the TCGA-COAD and GSE39582 study. For TCGA data, The TCGAbiolinks package was used to download the raw data of this cancer at the initial stage [9]. The edgeR package deleted zero or near-zero gene expression from the expression matrix based on the CPM (Count per million) criterion, in which the CMP was less than 10% in 50% of the samples. Following that, the data were normalized using the TMM approach and then transferred to a logarithmic state based on 2 by using limma package [10]. All analyses, including discovering differences in expression between groups and the link between TF expression and patient prognosis, were conducted using the generated expression matrix. There were 480 tumor specimens at various stages and 41 normal specimens in this study. The most recent TCGA-COAD clinical data update was retrieved and used in the study. For GSE39582, this study contained 566 colorectal tumor specimens and 19 normal colon specimens. The limma package performed the initial preprocessing of the GSE39582 data, including background correction, data normalization based on the RMA method, and logarithmic data transfer based on 2 [11]. Finally, the resulting expression matrix was used to examine the differential expression of candidate TFs.

### Clinical data preprocessing, prognosis and score risk calculation

The association between TFs level and survival rate in patients with CRC was investigated using TCGA-COAD clinical data. Normal specimens, specimens with a life expectancy of 1 or NA, and specimens with a death status without a tumor at the time of death were eliminated. To look into the link between TFs expression and patients’ prognosis, all TFs expression was first collected from samples with the clinical circumstances specified. The expression of each TF in all samples was then converted to a Z-score, and the association between TF expression and the patient prognosis was investigated using a univariate Cox regression test. Clinical parameters including age, sex, stage and TNM.T were considered. The following formula was used to calculate the risk score of patients based on the expression of TFs:

Risk score = Expression of TF1 * coefficient B related to multivariate test + Expression of TF2 * coefficient B related to multivariate test +….

### Co-expression network and database

The human transcription factor (HTF) database (http://humantfs.ccbr.utoronto.ca/) was used to derive a list of all TFs and their gene information. The co-expression network was used to find the pathways related to the discovered TFs. The expression correlation (Pearson) between the expression of each TF and all available genes was investigated using the normalized data matrix. Finally, the genes having the highest expression association with each TF (R > 0.6, *P* < 0.01) were chosen. All the genes in the co-expression network were enriched through the information of the MSigDB database by ErnrichR (https://maayanlab.cloud/Enrichr/) tool. In actuality, the pathways related to the candidate TFs were identified using the data from the mentioned databases.

### CRC sample collection

At Milad Hospital, thirty samples of tumors and adjacent normal tissue were surgically taken (Isfahan, Iran). A pathologist confirmed all of the cancer samples. Furthermore, the samples were obtained with the candidates’ consent and were approved by the ethics committee with the number of access IR.PNU.REC.1400.224. Table 1 summarized the clinical information from the samples obtained. Until they were used, all samples were preserved in liquid nitrogen.

**Table 1:** Clinical information for CRC samples

Cancer name	Characteristic	Number (N = 30)
**CRC**	**Age**	
	< 50 > 50	9 21
	**Gender**	
	Male Female	18 12
	**TNM stage**	
	I II III IV	5 11 9 5
	**Tumor size**	
	< 5 cm > 5 cm	12 18
	**Tumor site**	
	Right colon Left colon Cecum Sigmoid Rectum	6 7 2 10 5
	**TNM.N**	
	N0 N1	17 13

### RNA extraction, cDNA synthesis and RT-qPCR

All samples were washed three times with PBS- to remove necrotic tissue and contaminants. The TRIzol (Invitrogen) method was used to extract RNA, which was done according to the manufacturer’s instructions. After that, DNase (Fermentas) was used to remove any possible DNA contamination. cDNA was generated from the isolated RNAs using the TaKaRa cDNA synthesis kit. The primer-blast tool (NCBI) was used to design primers for the *FOXS1* (F: 5’-CCTGGAAGCTGAGCCTGACC-3’ and R: 5’-TAGCAATAAGGGCGATGTAGCTGT-3’) and *CASZ1* (F: 5’-ACCGTCTCCACTGTCAAGAACG-3’ and R: 5’-TCAGGGTCAAGGCAGTGGTAGT-3’) genes. SYBR Green PCR master mix (TaKaRa) and specific primers for each gene were used in RT-qPCR. The *GAPDH* (F: 5’-TGCCGCCTGGAGAAACC-3’, R: 5’-TGAAGTCGCAGGAGACAACC-3’) level was used as an internal control, and 2^−ΔCt^ was used to calculate the expression of each gene in each sample [12].

### Software and statistics

Using the R programming language (V 4.0.2), all the initial preprocessing were performed on the raw TCGA and GEO data, and the latest update of the mentioned packages was used. The linear model method was used to examine the difference in expression between the groups, and the FDR level < 0.01 was considered. The significance of the differences in RT-qPCR data between groups was assessed using the T-test, and the significance of the association between TF expression and patient survival was examined using the logRank test. All shapes and diagrams were drawn by GraphPad Prism (V 8) software and Cytoscape (V 3.7).

## Result

### The relationship between abnormalities in TF expression and patient survival

**Fig. 1:**
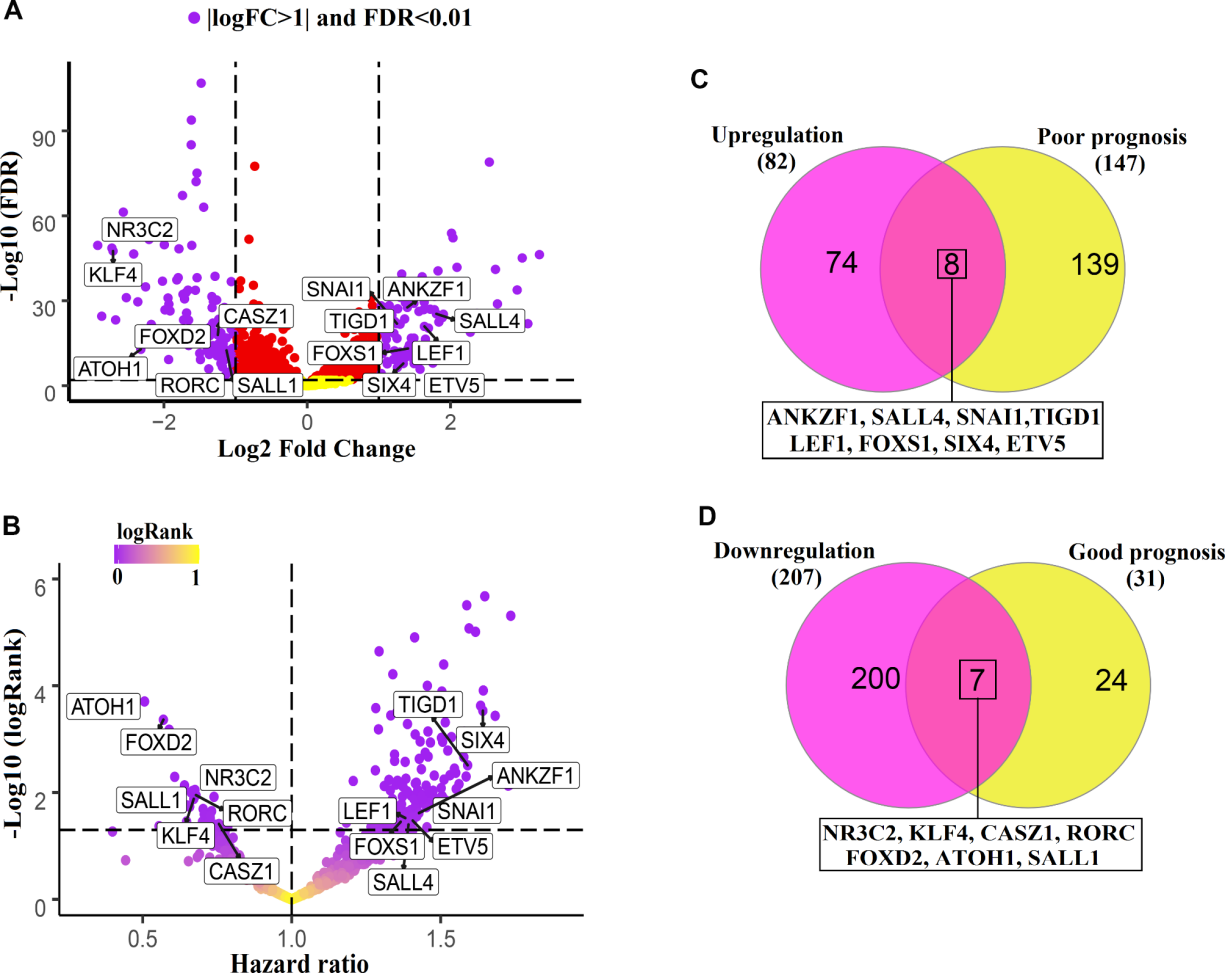
Increased and decreased TF expression is associated with the prognosis of patients with CRC. (A) Volcano plots for differential expression of all TFs in CRC compared to normal are illustrated based on TCGA data. Criteria | logFC |> 1 and FDR < 0.01 were considered for the selection of TFs that might play an oncogenic and tumor suppressor role. (B) Cox regression results for TFs expression level correlation with patients’ prognosis in CRC based on TCGA clinical data are shown. (C) A Venn diagram for common overexpressed (logFC > 1 and FDR < 0.01) and poor prognostic (HR > 1 and logRank < 0.01) TFs is illustrated. (D) Common TFs between down expressed (logFC< -1 and FDR < 0.01) and good prognostic genes (HR < 1 and logRank < 0.01) are shown.

For a better insight into changes in TFs expression in CRC, TCGA data were utilized, and the expression of all TFs was extracted from HTF database. Of the 1637 TFs in the HTF database, only 1467 TFs were expressed in normal and CRC tissues after the removal of zero-expression genes from the data. The results of expression difference showed that 82 TFs had increased in level, and 207 TFs had decreased in expression with |logFC|>1 and FDR < 0.01 (Fig. 1A). On the other hand, the relationship between the expression of expressed TFs and the prognosis of patients was assessed. The findings demonstrated that 147 TF levels were linked to bad prognosis (HR > 1, logRank < 0.01), while 24 of them were associated to good prognosis (Fig. 1B, HR < 1, logRank < 0.01). To identify genes with both oncogenicity and poor prognosis features, common genes between 82 overexpressed genes and 147 poor prognostic genes were selected, among which eight genes, including *ANKZF1*, *SALL4*, *SNAI1*, *TIGD1*, *LEF1*, *FOXS1*, *SIX4* and *ETV5* were recognized (Fig. 1C). *NR3C2*, *KLF4*, *CASZ1*, *FOXD2*, *ATOH1*, *SALL1*, and *RORC*, on the other hand, were linked to a considerable reduction in expression as well as a good prognosis in patients (Fig. 1D). These results suggest that these TFs may function as tumor suppressors and oncogenes, and they could also serve as useful biomarkers for the prognosis of CRC.

### Predicting the survival rate of patients with CRC based on the level of ***ANKZF1***, ***LEF1***, ***ATOH1*** and ***CASZ1***

**Fig. 2:**
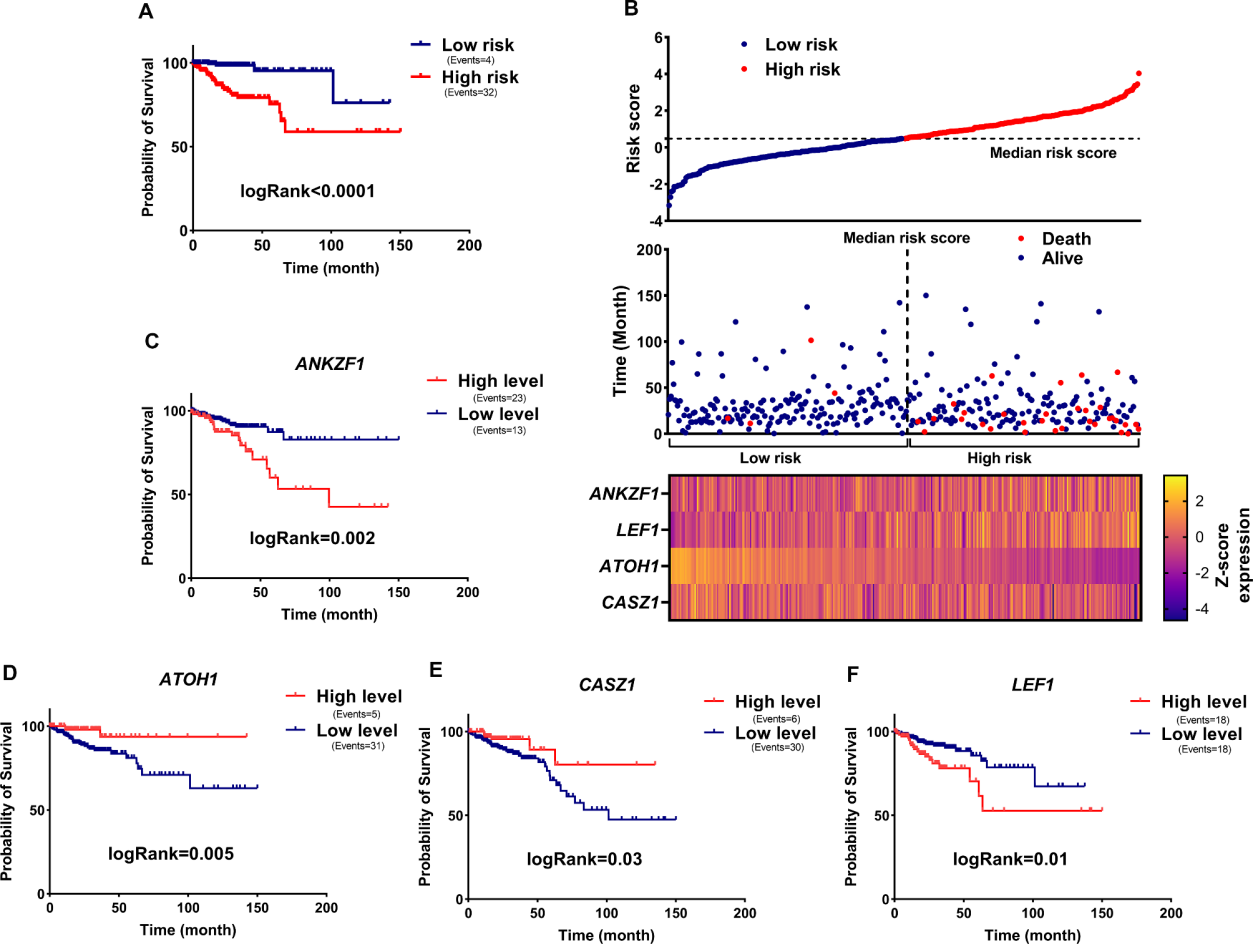
Expression of *ANKZF1*, *LEF1*, *ATOH1*, and *CASZ1* can predict patient mortality risk. (A) The Kaplan-Meier diagram is illustrated for high-risk patients versus low-risk patients. The expression mortality risk was computed based on the expression of *ANKZF1*, *LEF1*, *ATOH1* and *CASZ1*, and the risk score median was applied as cut-off value. (B) The scatter plot of *ANKZF1*, *LEF1*, *ATOH1* and *CASZ1* genes is depicted in relation to patient mortality. (C-F) The K-M plot is demonstrated for *ANKZF1*, *LEF1*, *ATOH1* and *CASZ1* in CRC samples based on TCGA data. Median expression was applied as the cut-off value for dividing patients into high expression and low expression groups.

The preceding phases revealed that the expression of some TF in CRC samples rose or reduced when compared to normal and their level was associated to patient prognosis. A multivariate Cox regression test was utilized to understand better the expression of TFs as prognostic markers and their independence from clinical characteristics. Only the expression of the *ANKZF1*, *LEF1*, *ATOH1*, and *CASZ1* TFs was found to be independent of clinical factors in predicting patients’ prognosis in multivariate analysis (Table 2, logRank < 0.05). In this case, each patient’s risk score was determined using the expression levels of the *ANKZF1*, *LEF1*, *ATOH1*, and *CASZ1* through the formula in the materials and methods section. The findings revealed that the expression of *ANKZF1*, *LEF1*, *ATOH1*, and *CASZ1* might be used to predict the mortality risk of patients (Fig. 2B). Kaplan-Meier results also confirmed the results and showed that high-risk patients had lower survival rates and prognoses (Fig. 2A, logRank < 0.01). To further confirm, TCGA cancer samples were divided two group (low and high) based on *ANKZF1*, *LEF1*, *ATOH1* and *CASZ1* expression medians and examined by Kaplan-Meier curve. The results showed that increased expression of *ANKZF1* and *LEF1* was associated with increased mortality, and increased expression of *ATOH1* and *CASZ1* was related with declined mortality of patients (Fig. 2C-F, logRank < 0.05). These results suggest that the expression of *ANKZF1*, *LEF1*, *ATOH1* and *CASZ1* can significantly predict the mortality rate of CRC patients and could have biomarker potential to predict the prognosis of CRC patients.

**Table 2:** Cox regression test results for identified TFs and related to patient survival in the presence of clinical features

	Univariate			Multivariate		
	HR	***P*** value	95% CI	HR	*P* value	95% CI
Age (60 > vs. <60)	1.8	0.12	0.92–3.39	-	-	-
Gender (Female vs. Male)	1.4	0.23	0.72–2.63	-	-	-
**Pathological Stage** **(P1-P2 vs. P3-P4)**	**11.9**	**2.4E-09**	**4.2–33.6**	**11.3**	**0.0008**	**3.37–18.23**
TNM T stage (T1-T2 vs. T3-T4)	9.3	0.007	1.27–21.32	1.76	0.59	0.21–14.67
***ANKZF1*** **expression** **(High vs. Low)**	**1.42**	**0.02**	**1.04–1.96**	**1.53**	**0.02**	**1.02–2.32**
*SALL4* expression (High vs. Low)	1.39	0.03	1.01–1.91	1.07	0.74	0.67–1.71
*SNAI1* expression (High vs. Low)	1.46	0.01	1.07–1.98	1.32	0.2	0.86–1.97
*TIGD1* expression (High vs. Low)	1.59	0.003	1.16–2.16	0.99	0.92	0.64–1.54
*FOXS1* expression (High vs. Low)	1.36	0.03	1.02–1.82	0.87	0.52	0.56–1.33
*SIX4* expression (High vs. Low)	1.64	0.0002	1.24–2.16	0.95	0.82	0.62–1.43
***LEF1*** **expression** **(High vs. Low)**	**1.38**	**0.03**	**1.03–1.85**	**1.65**	**0.01**	**1.1–2.43**
*ETV5* expression (High vs. Low)	1.4	0.03	1.02–1.92	1.16	0.56	0.69–1.96
*NR3C2* expression (High vs. Low)	0.67	0.008	0.51–0.9	1.31	0.23	0.64–1.43
*KLF4* expression (High vs. Low)	0.68	0.01	0.48–0.92	1.32	0.35	0.72–2.41
***ATOH1*** **expression** **(High vs. Low)**	**0.5**	**0.0001**	**0.35–0.72**	**0.51**	**0.01**	**0.29–0.89**
***CASZ1*** **expression** **(High vs. Low)**	**0.71**	**0.03**	**0.58–0.91**	**0.65**	**0.01**	**0.46–0.9**
*FOXD2* expression (High vs. Low)	0.56	0.0004	0.41–0.78	0.62	0.06	0.39–1.09
*RORC* expression (High vs. Low)	0.67	0.01	0.52–0.89	0.95	0.82	0.64–1.45
*SALL1* expression (High vs. Low)	0.55	0.03	0.41–0.97	0.72	0.14	0.47–1.11

### Alternation in ***FOXS1*** and ***CASZ1*** expression levels in CRC samples and association of metastatic genes with overexpressed TFs

**Fig. 3:**
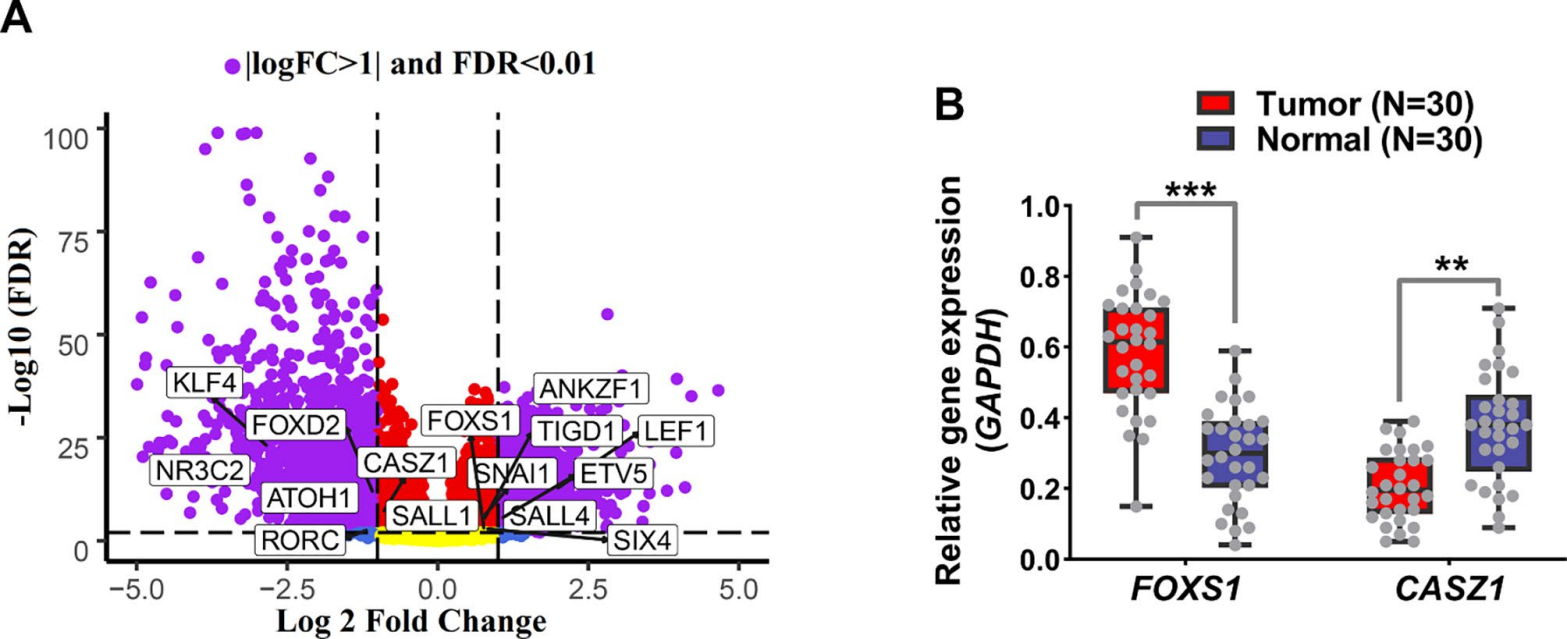
*FOXS1* and *CASZ1* had up and down expressions in CRC patients, respectively. (A) Differential gene expression results for candidate TFs is shown in GSE39582. All candidate TFs except *SALL1* had significant expression changes in line with previous results. (B) Expression differences for *FOXS1* and *CASZ1* in CRC samples compared with adjacent normal tissue by RT-qPCR. T-test was utilized to evaluate the significance level. The expression of each gene in each sample was calculated based on 2^−ΔCt^.

**Fig. 4:**
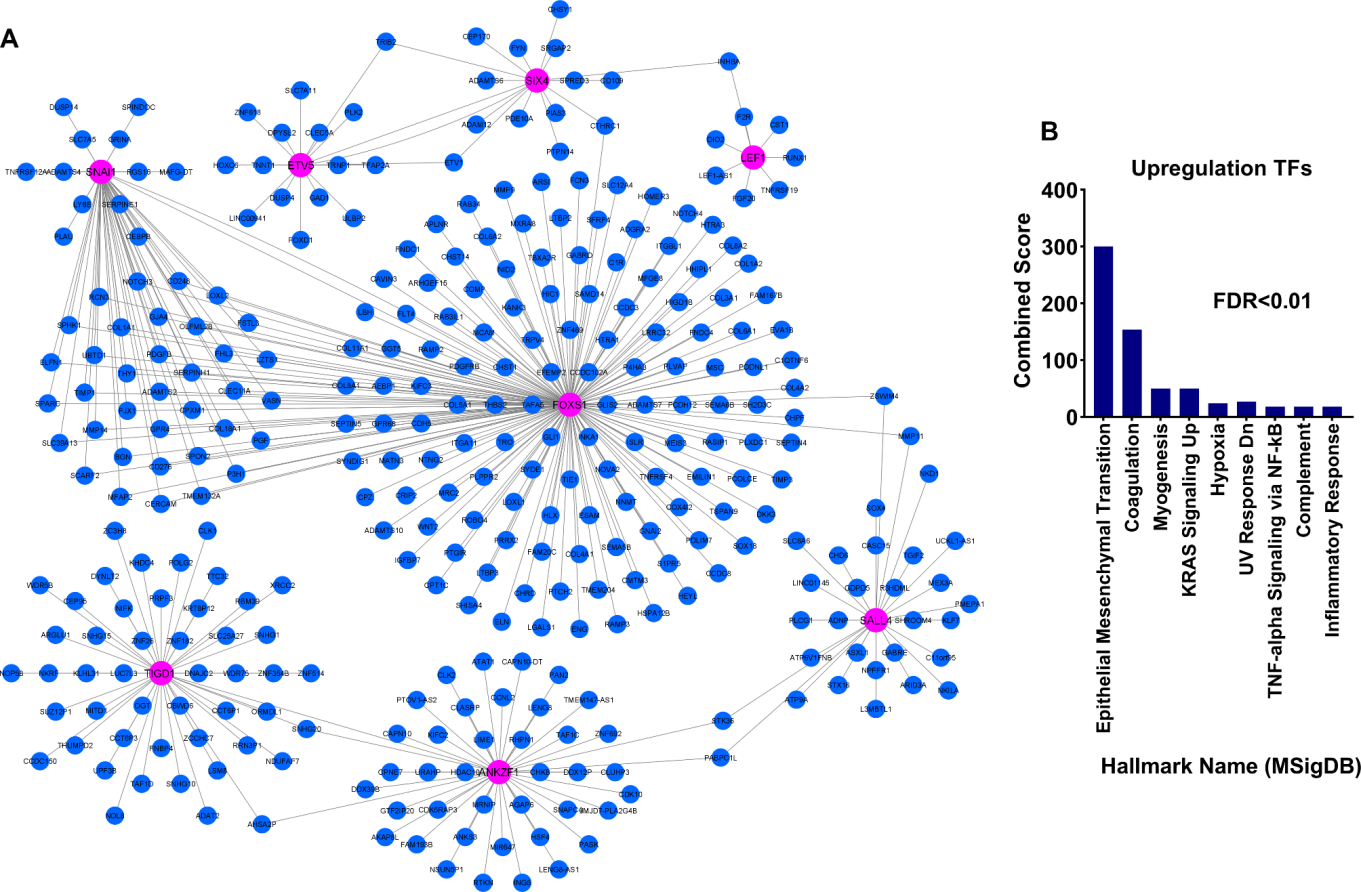
Overexpressed TFs were found to be strongly related to genes involved in cancer cell metastasis pathways. (A) Co-expression network for all candidate TFs based on TCGA data. Genes that had more than 0.5 correlation coefficient (R > 0.5, p < 0.01) with each of the candidate TFs were considered. (B) Enrichment results for genes that were significantly correlated with increased TFs and presented in the co-expression network.

**Fig. 5:**
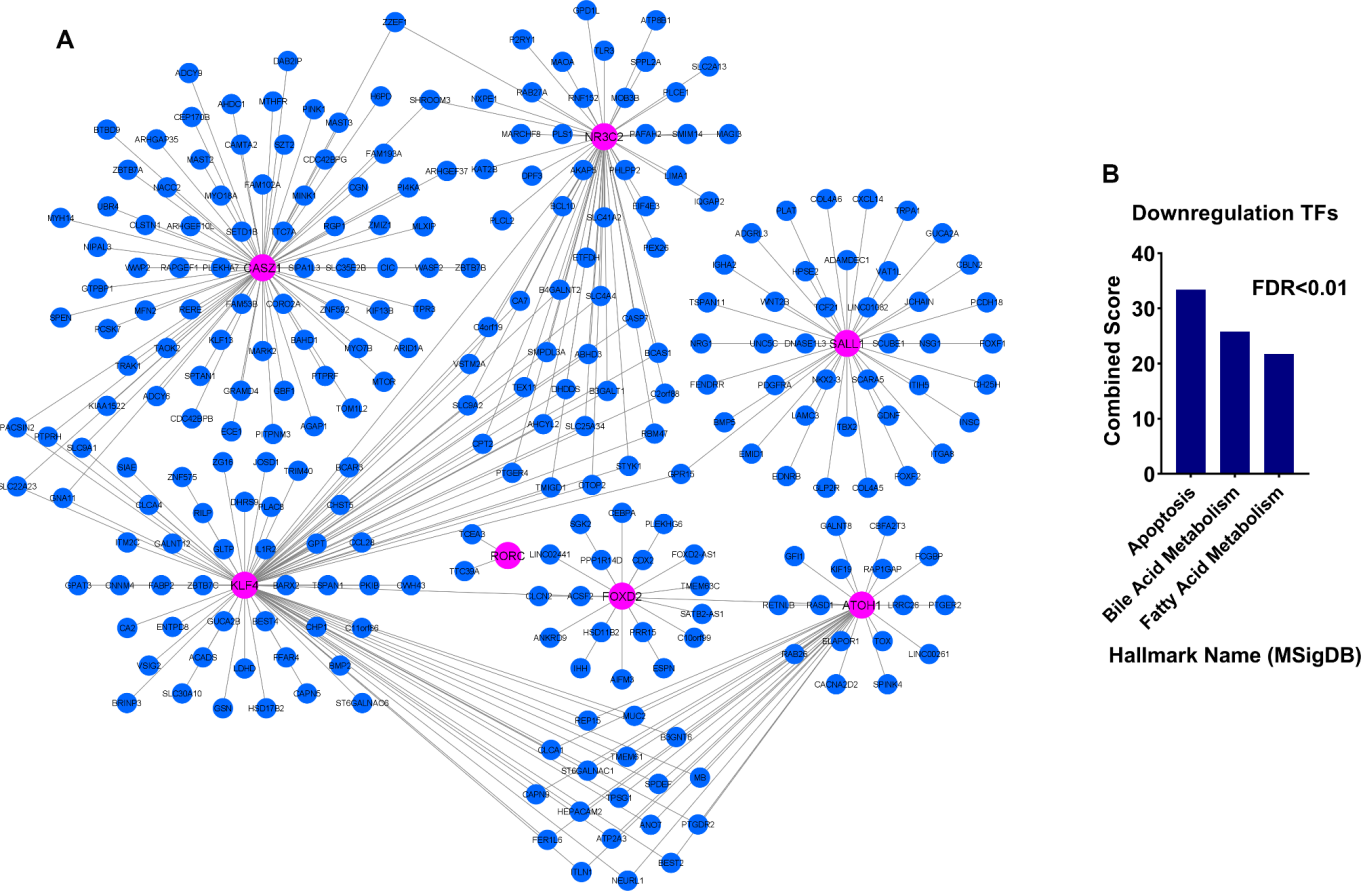
Decreased TFs with good prognosis are co-expressed with genes associated with the apoptotic pathway. (A) Co-expression network is shown for genes that are significantly associated with reduced TFs (R > 0.5, P < 0.01). (B) Enrichment outcomes of genes that were highly correlated with downregulated TFs and presented in the co-expression network.

The expression of TFs identified in the previous steps was investigated in GSE39582 to validate the acquired results further. The results showed that, except *SALL1*, all genes had consistent and significant alterations, which was consistent with earlier findings (Fig. 3A, FDR < 0.01). We used the CRC sample and the surrounding normal to assess the expression of the less well-researched *FOXS1* and *CASZ1* in CRC using the RT-qPCR approach in order to corroborate the earlier findings. *FOXS1* expression rose in TCGA and GSE39582 cancer samples, but *CASZ1* expression dropped, according to the results of the preceding steps. The expression of *FOXS1* in cancer samples increased considerably compared to adjacent normal samples, while the expression of *CASZ1* decreased (Fig. 3B, *P* < 0.01). In fact, RT-qPCR results confirmed the previous findings. The pathways involved in relation to the identified TFs were also identified through the co-expression network to understand their performance and roles better. As shown in the materials and methods section, the pathway analysis results showed that the overexpressed TFs were expressively correlated with the genes of the pathways associated with cancer cell metastases, such as epithelial mesenchymal transition and inflammation (Fig. 4A and 4B, FDR < 0.01). Decreased TFs, on the other hand, were associated with genes linked to the apoptotic pathway (Fig. 5A and 5B, FDR < 0.01). These findings imply that increased TFs may contribute to cancer cell metastasis in an oncogenic manner, whereas decreased TFs through apoptotic pathways may contribute to tumor suppression.

## Discussion

Many transcription factors (TFs) regulate gene expression and are involved in the pathogenesis of a variety of diseases, including CRC [13]. Because a TF’s activity and expression can alter the expression of a large number of genes, finding and understanding their role in disease is crucial. In fact, finding illness-related TFs can be used to more effectively target a wide number of genes involved in disease pathogenesis. Therefore, in this study, we discussed the role and expression of all TFs in CRC.

Our findings revealed that a vast number of transcription factors become dysregulated in CRC. The results of this study show that the expression of various transcription factors, including *ANKZF1*, *SALL4*, *SNAI1*, *TIGD1*, *LEF1*, *FOXS1*, *SIX4*, and *ETV5*, increased dramatically in CRC patients and was correlated with the poor prognosis. According to research, the expression of *ANKZF1, SNAI1, TIGD1*, and *LEF1* is up-regulated in CRC tissues and also is associated with a poor prognosis for patients [14–17]. On the other hand, it has been shown that *LEF1* can control the proliferation and invasion of CRC cells and play a significant role in the development and progression of CRC [18]. Additionally, it has been discovered that *FOXS1* can modulate EMT and cell proliferation in liver and stomach malignancies, which helps the diseases develop [19, 20]. For the first time, our findings revealed that *FOXS1* expression was higher in CRC samples than in normal samples, indicating that this transcription factor could have a role in the genesis and aggressiveness of CRC. *SIX4* also increases metastasis in CRC via the PI3K-AKT pathway [21]. The co-expression network of this study’s findings revealed that the pathways for EMT and metastasis are linked to genes associated with elevated TFs. We also showed for the first time that the expression of *ANKZF1*, *LEF1* independent of patients’ clinical features could predict the patient’s risk of death. In light of this, it is proposed that the *SALL4*, *SNAI1*, *TIGD1*, *LEF1*, *FOXS1*, *SIX4*, and *ETV5* as TFs may have carcinogenic potential in CRC.

Our findings revealed that the expression of TFs including *NR3C2*, *KLF4*, *CASZ1*, *FOXD2*, *ATOH1*, and *RORC* in CRC samples was much lower than in normal samples and that their increased expression was linked to the good prognosis of patients. Reduced *NR3C2* expression is related to increased invasion and proliferation of CRC cell lines [22]. *KLF4*, on the other hand, is demonstrated to decrease the proliferation of CRC cells and can play a tumor suppressor role in this malignancy, according to a study [23]. The results demonstrate that *CASZ1* can regulate gene expression to act as a tumor suppressor in neuroblastoma tumors [24]. Additionally, significant research has demonstrated that by limiting cell proliferation, *CASZ1* can contribute to the inhibition of tumor growth in a variety of malignancies [25]. It has been demonstrated that *ATOH1* has a tumor-suppressing function and that it is also decreased in CRC [26]. The results of this study also showed that *ATOH1* and *CASZ1* expression could predict CRC patient survival independent of clinical characteristics. We also discovered that *CASZ1* expression was considerably lower than in normal samples compared to CRC samples. The results of the co-expression network showed that decreased TF expression was associated to apoptosis-related genes. According to these results, the expression levels of *NR3C2, KLF4, CASZ1, FOXD2, ATOH1*, and *RORC* reduce in CRC and may function as tumor suppressors. The outcomes of this study indicate that the mentioned transcription factors can be potential therapeutic targets to treat and predict the survival of patients with CRC. One of the limitations of this work is that although the TFs discovered may contribute to the pathogenesis of colorectal cancer, these findings still require further in vitro and in vivo testing.

## Conclusion

The findings of this study revealed that the expression of many TFs changes in CRC significantly. The TFs expression of *ANKZF1*, *LEF1, CASZ1* and *ATOH1* are deregulated, which are associated with prognosis in patients. These TFs have the potential to be therapeutic targets as well as reliable biomarkers of CRC. We also showed for the first time that *FOXS1* and *CASZ1* expression are significantly altered in CRC, and we also suggest that these two transcription factors may have a role in the onset and progression of CRC.

## Data Availability

Supporting and raw data are available upon a reasonable request to the corresponding author.

## Abbreviations

TFs: Transcription factors
CRC: colorectal cancer
TCGA: the cancer genome atlas
HTF: the human transcription factor
