# Correction: The expression changes of transcription factors including *ANKZF1*, *LEF1*, *CASZ1*, and *ATOH1* as a predictor of survival rate in colorectal cancer: a large-scale analysis

**DOI:** 10.1186/s12935-023-02878-x

**Published:** 2023-03-24

**Authors:** Manizheh Sajadi, Mohammad Fazilati, Habibollah Nazem, Mohammad Mahdevar, Kamran Ghaedi

**Affiliations:** 1 Department of Biochemistry, Faculty of Science, Payame Noor University, Tehran, Iran; 2 Genius Gene, Genetics and Biotechnology Company, Tehran, Iran; 3 Department of Cell and Molecular Biology and Microbiology, Faculty of Biological Science and Technology, University of Isfahan, Isfahan, Iran

**Correction: Cancer Cell International (2022) 22:339** **https://doi.org/10.1186/s12935-022-02751-3**

In this original article [1], the affiliation for the authors Manizheh Sajadi, and Habibollah Nazem was incorrect. The correct affiliation is as follows “Department of Biochemistry, Faculty of Science, Payame Noor University, Tehran, Iran”.

Also, the correct academic email address of the author Dr. Fazilati should be mfazilati@pnu.ac.ir.
